# Audience response system (ARS); A way to foster formative assessment and motivation among medical students

**DOI:** 10.15694/mep.2021.000120.1

**Published:** 2021-05-11

**Authors:** Shazia Iqbal, Shahzad Ahmad, Khalid Akkour, Fatimah Taher AlHadab, Sana Hussain AlHuwaiji, Manal Abdullah Alghamadi

**Affiliations:** 1 Alfarabi College of Medicine Riyadh; 2 Alfarabi College of Dentistry Riyadh; 3 King Saud University Riyadh

**Keywords:** Audience response system (ARS), Fostering formative assessment, Interactive teaching strategies, Technology-enhanced learning, Teaching tools, Motivational tools, Engaging tools, Poll Everywhere, Pedagogical model

## Abstract

This article was migrated. The article was marked as recommended.

Using an audience response system (ARS) is very effective to improve learning through active participation and enhanced interaction among medical students. Although the validity of ARS in engagement during the learning process is clear, we know little about its impact on self-motivation.

**Aim:** This study aimed to investigate the effectiveness of ARS as compared to other used methods such as quizzes on paper/verbal questioning and analyzed how ARS nurture motivation among medical students.

**Methods:** This is mixed-method research and assessed the medical student’s perceptions about the use of ARS and its role in the augmentation of motivation for learning. We used a google doc questionnaire for data collection and the qualitative part of the study we conducted by semi-structured focused group discussions to explore the impact on self-motivation. For data analysis, we applied students paired t-test, and a p-value of less than 0.05 determined the significant correlation between ARS and other methods of formative assessment (quiz on paper/verbal questions). We coded the focused group discussions and identified themes based on the grounded theory.

**Results:** There is a significant correlation (P value= 0.008) for the comfort level of the formative assessment methods with ARS as compared to quizzes on paper/verbal response. ARS helped significantly to enhance the self-evaluation and self-motivation through instant comparisons of the results with the rest of the audience with P-value=0.027 and 0.021 respectively. ARS is a validated and proficient tool to assess student’s learning and generating self-motivation.

**Conclusion:** In medical education, ARS enhances the opportunity to recognize the knowledge gaps and encouraged conceptual learning. It provided students with a powerful insight into the learning of key concepts and self-assessment. Using ARS triggers not only motivation but also generates novel ideas and lifelong learning skills.

## Introduction

### Background

Currently, the use of groundbreaking technology in educational programs indicates significant progress in medical education. There is a significant development in the formative assessment with the practice of multiple educational technologies. Such as the use of an audience response system (ARS) is very effective to improve learning through active participation and enhanced interaction among students. ARS also is known as classroom voting systems/ electronic voting systems or personal response systems. It is one of the forms of an instant response system that provides each participant with a handheld input device or mobile phone through which they can communicate anonymously with software. The adoption of ARS provides feasibility and flexibility to conduct a formative assessment ( Hussain and Wilby, 2019). We consider formative assessment as a form of continuous evaluation used to assess the learning needs, comprehension of the subject by learners, and continuous academic progress during the teaching sessions.

The use of ARS can enhance a learner’s engagement in the learning process and boost the teaching efficiency. It is meant to engage the learner into conceptual learning and boost the satisfaction of medical education participants ( Atlantis and Cheema, 2015). There are various kinds of instant response systems that are being used in medical education; for example instant mobile audience response systems (mARS), Poll Everywhere, and Socrative, etc. Implementing cell phones used in the form of ARS made learning more versatile and affordable ( Mittal and Kaushik, 2020). The studies showed that the participants noticed an improvement in their attention span and a better understanding of topics with mARS during sessions ( Abdel Meguid and Collins, 2017; Beaumont *et al.,* 2017).

ARS promotes the quality of learning by increasing the interaction and improves the student’s learning outcomes ( Nelson *et al.,* 2012). ARS approach assists in instant data collection for reporting and feedback analysis after discussions ( Kay and LeSage, 2009). Besides, ARS has a significant role to augment the self-evaluation of learners ( Mostyn, Meade and Lymn, 2012). ARS has the potential for improvement activities about professional development because most participants stay alert and attentive. Few studies have reported a variety of benefits during conferences, social and engaging activities ( Grzeskowiak *et al.,* 2014).

### Problem

Medical educators are not only aiming for effective pedagogical tools but also struggling to develop tools that play an immense role in developing self-motivation and lifelong learning skills among medical students. The development of these skills encourages the learner to take up opportunities for their learning through self-evaluation and commitment to complete the task. ARS associated tools help to develop self-assessment, enhance self-awareness compared to the other participants of the session. Although many studies highlight the effectiveness of ARS in engagement in the learning transform, we know little about its impact on self-motivation and self-encouragement for learners. Additionally, there is a significant demand to know in-depth about the process of a learner’s self-motivation through ARS.

This research examined the effectiveness of ARS as a formative assessment tool in the comparison of other methods such as quizzes on paper/verbal questioning in the teaching sessions. It analyzed how ARS nurture motivation among medical students. Additionally, the authors investigated the process of ARS use as a formative assessment method through quantitative analysis. This article will help to teach faculty, medical educators, and the educational community to enhance the learner’s engagements during academic sessions.

**Figure 1:**
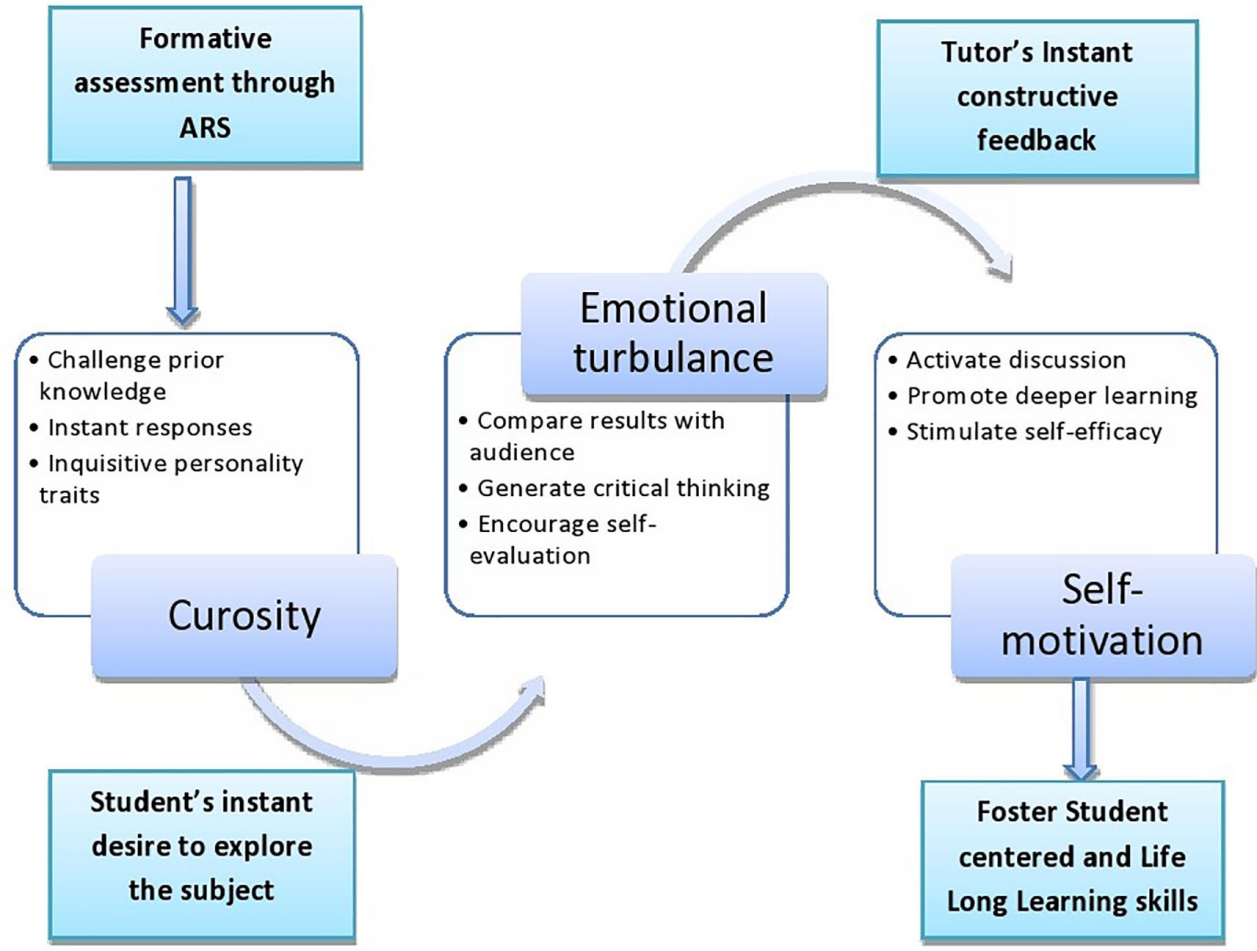
Process for the development of a learner’s self-motivation through audience response system

This study extracts the key factors which start curiosity among the learners; leading to emotional dissonance. Eventually, this mental state of learners leads to self-motivation and self-evaluation under influence of the immediate tutor’s constructive feedback. Figure 1 summarises the key elements influencing the development of self-motivation through-provoking curiosity and emotional conflict. During this process, students’ instant wish to explore the subject and prompt tutor’s feedback acts as a catalyst for the creation of self-motivation.

### Aim

This research aimed to explore the effectiveness of ARS as compared to other methods such as quizzes on paper/verbal questioning and analyzed how ARS nurture motivation among medical students.

## Methods

We used ARS during teaching and formative assessments over four years of the Bachelor of Medicine and Surgery (MBBS) program at the college of medicine from September 2016 to September 2019. Data was collected between October 2019 to December 2019. The study approached mixed methods for investigations and medical students of all years were provided prereading materials for interactive lectures (i-Lecture), team-based learning (TBL), and other teaching sessions such as tutorials/practicals. This material was provided through the Blackboard portal before the activities using the ARS and quizzes on paper/verbal questioning. They were randomly assessed by different formative assessment tools throughout the study period. A questionnaire was given to all participants for 30 min after teaching sessions.

### Quantitative Data Analysis

In the first part of the study, we conducted a quantitative analysis by establishing a structured questionnaire and collected data through google doc. A study survey was adjusted from a authenticated questionnaire established by Pierce ( Gubbiyappa *et al.,* 2016). We modified questionnaire and confirmed in the pilot study appropriately to suit the needs.

### Study Instruments

We used “Poll Everywhere” software for polling and feedback.

### Inclusion Criteria

All the undergraduate students of the Bachelor of Medicine and Surgery (MBBS) program at the college of medicine from September 2016 to September 2019. were included. All students were randomly exposed to ARS and quizzes on paper/verbal questioning activities.

### Exclusion Criteria

Incomplete quiz were omitted from the analysis of results.

### Data Analysis

For data analysis, we applied a student’s paired t-test to determine the correlation between ARS and other methods of formative assessment. To study the independent effects, the multiple logistic regression analysis was performed for each variable. A p-value of lower than 0.05 indicated the significant link between attributes such as self-evaluation, self-motivation. This part of the study helped to set up a strong relationship between the development of self-motivation through ARS.

### Qualitative Data Analysis

In the second part of the study, we conducted focused group interviews from six groups of students and different levels of the MBBS program. Each group had five to seven students with average GPA scores. These students had continuous exposure to ARS formative assessments and other methods of formative assessments (quizzes on paper/verbal questioning) during four academic years. The semi-structured questions were applied to know the perception of students about ARS. We aimed to focus on the process and impact of ARS on self-motivation for learning. questionnaire apprehended and analyzed mentioned the following items. The Semi structures questionnaire mentioned the following items:

• How about your experience of formative assessment activities assisted with the use of ARS as compared to other modes of assessments?
• How about your perceptions about the impact of ARS on self-motivation?
• How does the use of ARS enhance motivation?
• How the use of ARS help you to deeply engage in the study process?
• How does the use of ARS impact your self-evaluation?

The same cohort of students was included in this component of the study. The sample size was not fixed, and we took an iterative method of concurrent data collection until reached data saturation. We carried a thematic analysis by using NVivo and varified the qualitative data by two experts for validity. We coded the focused group discussion and identified themes built on grounded theory. We guaranteed participants to retain their identity unknown.

## Results/Analysis

In the study, 170 students took part in filling the survey out of 200 expected participants, and the response rate was 85% in quantitative analysis. Regarding the demographic data, the female response was more than males. Most participants were Saudi national and final year of the MBBS program as shown in Table 1.

**Table 1:** Demographic characteristics of study participants

Characteristics of participants	Sample	
	N=170	Percentage
**Gender**		
**Female**	98	57.6
**Male**	72	42.3
**Year of study**		
**Second year**	4	2.3
**Third Year**	12	7
**Fourth Year**	20	11.7
**Fifth Year**	58	34.1
**Final Year**	76	44.7
**Saudi National**	159	93.5
**Non-Saudi National**	11	6.5

**Figure 2:**
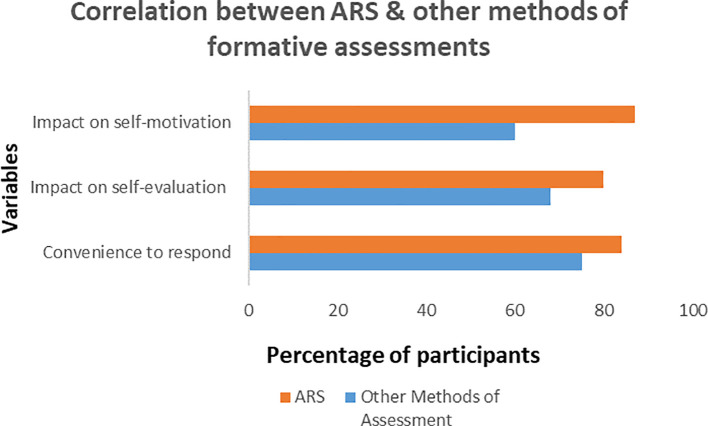
Comparison between formative assessments through ARS and other methods (quizzes on paper/verbal questioning)

We studied the impact of ARS on convenient methods for formative assessment, self-evaluation, and self-motivation as shown in Figure 2. The analysis found that there are significant correlations (P value= 0.008) for the comfort level of the formative assessment methods with ARS as compared to quizzes on paper/verbal response. It related the comfort level to the convenience of tool usage, efficacy, and efficiency of the assessment method. The study found that ARS helped significantly to enhance conceptual learning, self-evaluation, and self-motivation through instant comparisons of their results with the rest of the audience with P-value=0.027 and 0.021 respectively as shown in Table 2.

**Table 2:** Correlation between formative assessments through ARS and other methods (quizzes on paper/verbal questioning)

Summary of Items	*Coefficients*	*Standard Error*	*t Stat*	*P-value*	*Lower 95%*	*Upper 95%*	*Lower 95.0%*	*Upper 95.0%*
**Convenience to respond**	0.119	0.044	2.678	0.008	0.031	0.208	0.031	0.208
**Impact on self-evaluation**	0.103	0.046	2.225	0.027	0.011	0.195	0.011	0.195
**Impact on self-motivation**	0.105	0.051	2.050	0.021	0.003	0.206	0.003	0.206

While focused group interviews help to know the process of enhancement. It determined that curiosity during polling and emotional turbulence to know the right answer instantly is a triggering factor to boost self-confidence and self-motivation. Most participants agreed that responding to ARS questions during lectures, tutorials, and immediately seeing the distribution of responses gave them an insight that how should they study further to improve the subject as shown in Table 3. It helped participants to know the weak part of the subjects and help to improve by the focused study.

**Table 3:** Thematic analysis to develop self-motivation through use of ARS

No	Themes	Direct participant quotes and responses during interviews
1	**Curiosity development**	• Looking at the answers immediately, I could compare my response with the rest of the class. • I could immediately find my knowledge about the topic compared to other classmates. • I had realized that my attention and engagement in the session was more when the teacher uses ARS for a surprise quiz. • I never missed -----classes because the tutor asked questions interestingly and I feel convenient to answer. It’s grand fun. • ARS sessions always stimulated my brain and generated a sense of competition, which driven me to learn more and more. • One has to be curious about personality to go along with technology. • I always enjoyed the ice breakers’ images and questions posted through ARS.
2	**Emotional turbulence**	• I might not have answered two questions verbally as I feel nervous in front of my classmates. • I was feeling stress about my wrong answer and was disturbed emotionally. However, the tutor’s immediate feedback made me realize that how I can improve the further study. • Although I feel down sometimes, a session with ARS helped me to resolve my conflicts and misconceptions compared to my peers. • It was not only difficult for me, but my colleagues were also facing problems in asking questions because of poor English. • Once I recognized the right answer, but I didn’t feel comfortable raising a hand as I feel shy talking in the audience. • I feel envy that my colleagues knew the answer, and I didn’t know. That terrible feeling promoted a sense of self-study and in-depth learning.
3	**Self-motivation**	• I feel motivated after tutor instant feedback. • My brain started probing after active discussion during the case base session (Clinical scenarios) that why this topic was important and how could I grab more knowledge. • Discussion generating questions was very engaging as gave more chances to explore the variety of options in clinical scenarios. • After the discussion, our team presented novel ideas to manage a tough situation. • Empowerment and reassurance by the tutor encouraged me to study more about the subject. • We think dividing sessions into teams and creating competitions among groups is highly motivating because we always struggle to win prizes at the end. • I could study in depth because I found my knowledge poor as compared to my colleagues. • A blend of team-based learning (TBLs) and ARS was amazing as our team won the award. • We were confident to perform in final exams after ARS supported formative assessment.

## Discussion

Medical students found ARS a vital and effective means for deep learning as compared to other methods of formative assessment (quizzes on paper/verbal questioning). They perceived that ARS not only helped them to find their learning needs but also instigated them the specific learning objectives that required more attention. They developed critical thinking skills and attentiveness during sessions. Instant responses and instant feedback from tutor augmented meaningful learning and triggered an internal wish to improve the learning. But tutors need to prepare sessions according to learning needs and immense responsibility lies over tutors to encourage participants for interactive discussion. The quality of discussion provoking questions and organization of ARS assisted sessions are the key elements for the successful engagement of learners. The students appreciated the interspersed use of ARS more than the pre or post lecturing usage.

### ARS as a convenient method of formative assessment

The study determined that ARS promotes a more positive and interactive learning environment ( Satheesh *et al.,* 2013). The students described ARS usage as a flexible, promoting, and stimulating way of teaching and formative assessment. Other common methods such as paper quizzes need resources in terms of paper usage, faculty time for marking papers, anxiety related to waiting time, and test scores at students’ end. Most students were comfortable using these electronic polling systems (Poll Everywhere), and because of their use, they noticed significant changes in learning during final assessments ( Llena, Forner, and Cueva, 2015). This refers to self-perceived scores on final assessments. In classroom environments, ARS has enhanced their cognitive abilities, critical thinking, and promoted learning experiences ( Gubbiyappa *et al.,* 2016; Leeds *et al.,* 2018). Because of immediate questioning and understanding of key points at once helps the learners to resolve misconceptions and develop a conceptual framework ( Cox, 2019).

The students with poor English language and reluctant to talk in the audience can find an easy means for the expression of their thoughts ( Wenz *et al.,* 2014). The study concluded that ARS technology can highlight the topics which need priority to focus and enhance learning during lectures ( Leung *et al.,* 2013). Even the less talkative students involve in the discussion and express their opinions.

Students mentioned that this way of the assessment helped to reduced exams associated with anxiety ( Lymn and Mostyn, 2010). Besides, significant enhancements in the last exam results were noted. Most of the participants revealed that ARS was an impressive measure of learning in teams and much better than the conventional audio-visual presentation.

### Impact on self-motivation

The sense of competition in the learning environment is a natural phenomenon for survival. The intellect of competition generated with the help of ARS brings the ultimate wish to move on and bring out the full potential of learners. Using ARS has been well-accepted and students believe that their performance is enhanced by these instant response devices ( Oliveira-Santos *et al.,* 2018). Our study determined positive relations between the use of ARS to the higher attendance rate and greater scores in final assessments ( Koval, Kim, and Makhlouf, 2020).

The investigation showed that the application of ARS to be operative in facilitating the debates during sessions, clinical evaluations, and enhance academic attitudes ( Metz *et al.,* 2016). The practice of formative assessment in the completion of educational tasks in large group classes enhanced team working capabilities ( Schlegel and Selfridge, 2014). Students build their collaborative skills, communication skills, interpersonal interactions, and generate a healthy learning environment ( Castillo *et al.,* 2020; Pace *et al.,* 2016). During the TBL sessions, ARS allowed the students to get immediate test scores of the teams and evaluation compared to other participants in the discussions. Immediate tutor’s feedback during sessions acts as a catalyst to provoke deeper learning. Besides, the combination of TBL and ARS can improve a learner’s motivation and inspiration to do more hard work ( Fujikura *et al.,* 2013).

### Research Gaps

Recently there is a trend towards game-based interactions in large group sessions to promote active participation during learning. There are requirements to promote game-based learning by maximizing the value of instant response systems and other technology tools. It requires further research to combine image-based educations in a large audience compared to questions based on formative assessment through ARS ( Richardson *et al.,* 2020). The bidirectional use of the audience response system will give us an even better insight into how and what our learners know ( Richardson, 2014).

## Conclusion

In medical education, the use of ARS is a very operative and proficient way to assess student’s learning. The student’s feedback determined that this system enhanced their opportunity to recognize the knowledge gaps and encouraged conceptual learning. It provided them with a powerful insight into the learning of key concepts and self-assessment. Using ARS triggers not only motivation but also generates novel ideas and lifelong learning skills.

Medical students found ARS an invaluable and effective means for deeper learning and self-motivation. Activating the nous of competition through ARS is not only aiding to enhance self-evaluation but also has a great impact on the development of self-motivation. ARS creates a state of curiosity by instant questionings and brainstorming. Being in this temporary phase of dissonance triggers learners to engage in multiple learning resources. The sense of accomplishment of one task promotes a vicious cycle to bring further impetus and success to grasp the subject. But, during this process tutors, constructive feedback acts as a catalyst to encourage learners for wider engagement in learning processes. This powerful enthusiasm generates an influential encouragement in mind, and spirit; which assists the learner to touch the peak of learning.

## Take Home Messages

• The audience response system (ARS) promotes dynamic learning among the participants, ensuing the improved performance during formative assessments.
• Medical students found ARS an invaluable and effective means for deeper learning and self-motivation.
• ARS assisted formative assessment provides the students with a powerful insight into the learning of key concepts and self-assessment.
• ARS for medical students enabled instructors to engage the learners and help them towards self-directed learning.
• Through ARS student-teacher interface is an enchanting vigor in cultivating student interest in learning.

## Notes On Contributors

**Dr. Shazia Iqbal** is working as an assistant professor and director of the medical education unit at Alfarabi College of Medicine, Riyadh, Saudi Arabia. She is the author of this article and developed the study concept/design. She assists in the undergraduate curriculum development with a special interest in innovative educational technologies in medical education. ORCID ID: https://orcid.org/0000-0003-4890-5864

**Dr. Shahzad Ahmad** is an associate professor Dental department, Alfarabi College of dentistry Riyadh, Saudi Arabia. He is co-author of this manuscript and has a keen interest in advanced technologies in health education.

**Dr. Khalid Akkour** is an assistant professor and chief Obstetrics and gynecology department College of Medicine, King Saud University, Riyadh. He is co-author of this manuscript and has a keen interest in different educational technologies and robotics.

**Dr. Fatimah Taher AlHadab** is a medical intern at Alfarabi college of medicine Riyadh. She is the co-author of this article and her area of interest is the role of educational technologies in medical education.

**Dr. Sana Hussain AlHuwaiji** is a medical intern at Alfarabi college of medicine Riyadh. She is a co-author of this article and her main contribution is the analysis of the study.

**Dr. Manal Abdullah Alghamadi** is a medical intern at Alfarabi college of medicine Riyadh. She is the co-author of this article and her area of interest is the role of educational technologies in medical education.
