# Supplementary material for: Audience response system (ARS); A way to foster formative assessment and motivation among medical students
Source: MedEdPublish (2016). 2021 May 11;10:120. [Version 1] doi: 10.15694/mep.2021.000120.1 (PMC10939530; doi:10.15694/mep.2021.000120.1)
Supplement: Peer review history

# Reviewer report: Reviewer response for version 1

**DOI:** 10.21956/mep.18930.r27069

**Published:** 2021-06-10

**Authors:** David Taylor

**Affiliations:** 1 Gulf Medical University, Ajman, UAE

This review has been migrated. The reviewer awarded 4 stars out of 5 Audience response systems are widely used, and there are several publications about their effectiveness – the reference list to this paper is a good entry into the literature for those new to it. The element of this paper that I find most interesting is the thematic analysis (results given in Table 3). The authors do not say whether the focus groups were recorded in some way, or whether the utterances were transcribed, but I am aware that it is possible to analyse both video and audio tracks directly in NVivo in more recent versions of the software. If this were simply a thematic analysis study, we would probably expect to see a little more analysis of the process. I expect that the direct quotes are examples drawn from a larger set of utterances.In considering table 2, I find myself wondering whether it was wise to conflate “quizzes on paper” and “verbal questioning”. It would be interesting to know, for instance, if ARS are more similar to written quizzes or to verbal questioning. As it stands we can only assume that any difference is down to the safety blanket on anonymity. For completeness I would really like to see a simple description of the columns giving the correlation for upper 95% and lower 95%. The wildcard in the pack for both arms of the study is the way in which the tutor led the discussion of the results, I would certainly have looked at the qualitative data to see what students said about their responses to the way the tutor asked the questions and responded to the data.This paper is interesting, and with a few small changes to explain the issues I mention above, I think it would repay reading by anyone working in this field, or considering using audience response systems – which are, of course available with the major on-line video conference platforms.

Reviewer Expertise:

NA

No decision status is available

# Reviewer report: Reviewer response for version 1

**DOI:** 10.21956/mep.18930.r27070

**Published:** 2021-05-13

**Authors:** P Ravi Shankar

**Affiliations:** 1 American International Medical University

This review has been migrated. The reviewer awarded 4 stars out of 5 This is an important and interesting study about the use of an audience response system (ARS) in a medical college in Saudi Arabia. ARS has become more user-friendly and cheaper with the use of multiple platforms which can be accessed using mobile phones and tablets. There are several platforms like Poll Everywhere, Kahoot, Trello, Word Cloud, Socrative, and Slido among others. Before one had used to use specialized devices and clickers for ARS. The authors have not mentioned ethical approval and informed consent. Figure 1 has some spelling errors which can be corrected. The authors can explain better the quantitative part of the study and Table 2. I am not sure whether Poll Everywhere which is a proprietary name is a suitable keyword. The anonymous nature of the responses, the excitement of participating in an activity, and the ability to know the correct response immediately are among the strengths of ARS. The quality of written English needs improvement at many places. All health science educators may find this study to be of interest.

Reviewer Expertise:

NA

No decision status is available
